# Beneficial bile acid metabolism from *Lactobacillus plantarum* of food origin

**DOI:** 10.1038/s41598-020-58069-5

**Published:** 2020-01-24

**Authors:** Roberta Prete, Sarah Louise Long, Alvaro Lopez Gallardo, Cormac G. Gahan, Aldo Corsetti, Susan A. Joyce

**Affiliations:** 1 University of Teramo, Faculty of Bioscience and Technology for Food, Agriculture and Environment, 64100, Via Balzarini 1, Teramo, Italy; 2 APC Microbiome Ireland, University College Cork, Cork, Ireland; 3 School of Biochemistry and Cell Biology, University College Cork, Cork, Ireland; 4 School of Pharmacy, University College Cork, Cork, Ireland; 5 School of Microbiology, University College Cork, Cork, Ireland

**Keywords:** Biological techniques, Microbiology, Environmental sciences, Biomarkers

## Abstract

Bile acid (BA) signatures are altered in many disease states. BA metabolism is an important microbial function to assist gut colonization and persistence, as well as microbial survival during gastro intestinal (GI) transit and it is an important criteria for potential probiotic bacteria. Microbes that express bile salt hydrolase (BSH), gateway BA modifying enzymes, are considered to have an advantage in the gut. This property is reported as selectively limited to gut-associated microbes. Food-associated microbes have the potential to confer health benefits to the human consumer. Here, we report that food associated *Lactobacillus plantarum* strains are capable of BA metabolism, they can withstand BA associated stress and propagate, a recognised important characteristic for GIT survival. Furthermore, we report that these food associated *Lactobacillus plantarum* strains have the selective ability to alter BA signatures in favour of receptor activation that would be beneficial to humans. Indeed, all of the strains examined showed a clear preference to alter human glycol-conjugated BAs, although clear strain-dependent modifications were also evident. This study demonstrates that BA metabolism by food-borne non-pathogenic bacteria is beneficial to both microbe and man and it identifies an evolutionary-conserved characteristic, previously considered unique to gut residents, among food-associated non-pathogenic isolates.

## Introduction

Food-associated microbes are proposed to interact with the human host and provide potential benefits to gut health^1,2^. However, it has proven difficult to determine if these benefits are related to the dietary components alone, a direct influence of foods upon the host microbiota, the microbes they feed or in tandem with effects elicited by the microbes that they carry, since few studies have examined the dissemination of microbes of food origin^1–3^. In order to survive GI transit and persist in the gut, resident and transient microbes must be equipped to endure dynamic physiological stresses. Following oral ingestion, bacteria encounter a number of human defence systems that are associated with secretions, among them bile. Bile is a digestive, gall bladder secretion, synthesized from cholesterol in the liver and recycled to it, in the terminal ileum^4,5^. Bile has a major role in the emulsification and solubilisation of lipids and it generates bile flow to facilitate hepatobiliary secretion of lipids, endogenous metabolites, and xenobiotics^6^. The major component of bile is bile acid (BA), representing >50% of the organic components, it acts as a digestive surfactant to present and emulsify dietary lipids and lipid-soluble vitamins for digestion and uptake^7^. Because of their detergent properties certain BA moieties, at high levels, have antibacterial and inflammatory properties including disruption of bacterial and host cell membranes, protein denaturation, iron and calcium chelation and they can cause oxidative damage to DNA^7^. Therefore, the ability of microbes to tolerate bile and BAs is recognised as important for their survival and for their persistence in the GI tract^8^.

In the liver, cholesterol is converted into primary BAs, chenodeoxycholic acid (CDCA) and cholic acid (CA), then conjugated, as N-acyl amidates, mainly to amino acids taurine or glycine and self-assembled into fat presenting micelles in the small intestine^9^. The intestinal microbiota, particularly *Lactobacilli* spp. can use bile acids as environmental signals and, in certain cases, as nutrients and electron acceptors^10^. Importantly, intestinal bacteria have a major role in bile salt metabolism^11,12^ mainly through microbial bile salt hydrolase (BSH) enzymes that catalyse bile salts deconjugation by hydrolysis of the amide bond to release the glycine/taurine moiety from the steroid nucleus^9,13,14^. This function is recognised as the crucial gateway reaction in BA biotransformation^9,14^, regenerating primary free BA CA and CDCA, to facilitate microbial formation of secondary bile acid deoxycholic acid (DCA) from CA, lithocholic acid (LCA) and ursodeoxycholic acid (UDCA) from CDCA as well as a range of intermediates (reviewed by Long *et al*., 2017). Many of these are now recognised as potent cell signalling molecules involved in regulating host lipid and cholesterol, glucose, and energy metabolism, drug metabolism, and the modulation of immune response^15,16^. These receptors include BA ligand-activated nuclear receptors, the farnesoid-X-receptor (FXR) in the liver and intestine which controls BA homeostasis, the vitamin D receptor (VDR) which has a number of different roles, as well as the energy homeostasis G protein-coupled bile acid receptor TGR5^16^.

Amino acids released by deconjugation can be further utilized as carbon and nitrogen sources for bacterial sustenance and survival^17^. BSH enzymes may also promote cholesterol integration into bacterial membranes to alter membrane potential, fluidity and tensile strength^18^. This is proposed to effectively improve the viability of intestinal bacteria^4^ by enhancing bile salt tolerance and sensitivity to host defensins. It has been suggested that BSH enzymes might represent detergent shock proteins that enhance *Lactobacilli* GI survival^19^. In support of this, strong correlation between microbial habitat and BSH activity is reported^8,20^. Furthermore, intestinal bile salt deconjugation is essential for intestinal homeostasis by influencing the size and the composition of both the bile acid pool and the intestinal microbiota^21^.

BSH deconjugation activity is well represented among intestinal microbial genera^10,22^, it is predominantly characterized among gram-positive species *Bifidobacterium*^23–25^, *Clostridium*^26,27^, *Enterococcus*^28,29^, *Listeria*^30^, and *Lactobacillus*^31–38^, including *Lb*. *plantarum* species^39–43^. In addition, several investigators reported that bile tolerance is a strain-specific trait and tolerances of species cannot be generalized within a species or a genus^15,16^. Early reports assigned BSH activity to GI associated commensals, but not to environmental isolates^8^. They found that *bsh* coding sequences were present among all of the major phyla and that BSH enzyme activity differs between environments. Where examined in their study, all gut associated *Lactobacillus* strains were capable of both glyco and tauro deconjugation of bile acids. In contrast, *Lactobacillus* isolated from fermented milk show a preference for glyco conjugated bile acids, although some overlap for tauro deconjugation (specifically TDCA) was detected for a number of strains^44^.

BA metabolism, through BSH activity is an important EFSA criteria for probiotic strain selection^45^. It is taken as an indication of the strains potential to survive gastro-intestinal transit. More recently microbial BSH activity was shown to influence microbe-host dialogue, to functionally regulate host lipid metabolism, to alter cholesterol metabolism and potentially influence weight loss^21^.

The exact mechanism by which BA signatures impact weigh loss is currently unclear, therefore, regarding BSH enzymes, a generalized rule cannot be applied. The importance of bile acid metabolism has been studied only in gut-associated microbes^4^. In this work, food-borne *Lb. plantarum* isolated and characterized by the authors^46,47^ as well as known type strains, were examined and shown to vary in their ability to endure bile-associated stress *in vitro*. In addition, the specific ability of each strain to metabolize bile acid moieties was examined. Since bile acid derivatives are being recognized as therapeutic agents for treating human metabolic diseases^19^, this work suggests that the properties of food-associated microbes may also provide benefits to the host and it reinforces a role for food-based microbes as important targets for consideration and development in functional foods.

## Results

### Potential for bacterial survival in GIT environment upon bile stress

#### Bile salt resistance and tolerance

**Figure 1:**
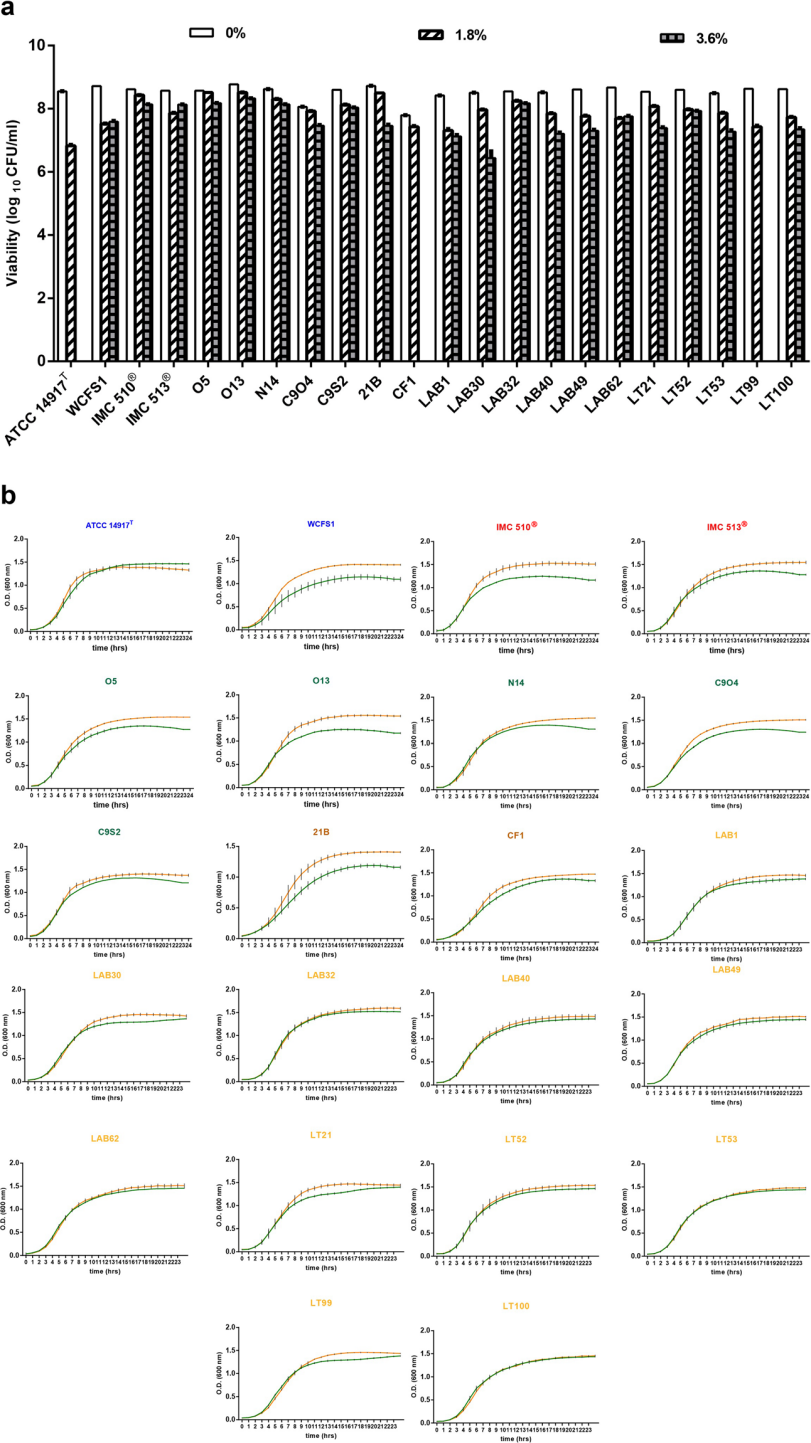
Food associated *Lb. plantarum* strains are resistant to bile salts. (**a**) The viability of *Lb. plantarum* strains was assessed on exposure to bile salts. Data are represented as mean values and bars indicate the standard deviation (SD) obtained from three replicates. Data were analysed by One-Way Anova followed by Bonferroni’s multiple comparisons post hoc test (0% vs 1.8% p < 0.05, 0% vs 3.6% p < 0.001). (**b**) Food associated *Lb. plantarum* strains are capable of anaerobic growth in the presence of bile. *Lb. plantarum* strains were assessed for growth under anaerobic conditions in the presence and absence of bile. Orange line indicates MRS broth alone, Green line indicates MRS broth supplemented with 0.5% porcine bile (0.5% w/v). Data are represented as the mean with standard deviation (SD) from the mean for three biological replicates.

The ability to grow in the presence of bile salts was monitored by measuring optical density at 600 nm during co-incubation with increasing concentrations of bile salts (0–3.6% w/v). At tested concentrations below 1.8%, microbial viability was unaltered (Fig. 1a and SI Figure S1a) for the majority of strains, however, significant reductions in viability were recorded with type strains *Lb. plantarum* ATCC14917, CF1 and LT99 isolated from pickled cabbage, sourdough, raw-milk cheeses respectively. The effect is more pronounced for these strains in the presence of 3.6% bovine bile where no viable growth is recorded for these 3 strains all of food origin (Fig. 1a). The remaining strains of food origin (olives, pickled cabbage, sourdough, raw-milk cheeses) could tolerate bile levels to the same extent as the human isolates WCFSI (from human saliva), IMC510, and IMC513 (human gut isolates). These data suggest a strong ability of food-borne *Lb. plantarum* strains to endure bile salt stress in concentrations higher than that found in the human gut to a similar extent to gut commensal *Lb. plantarum* representatives.

Considering that bile salts form micelles with phospholipids (as they are found in whole bile) and, that they may have lower antibacterial activity than artificial solutions of pure bile salts^48^, we investigated bile tolerance by incubating all the strains in MRS broth with 0.5% w/v porcine bile (of gallbladder origin) and monitored bacterial growth hourly over 24 hours (Figs. 1b and SI Figure S1b). All of the strains tested display a high ability to grow and survive during exposure to bile, both under aerobic and anaerobic conditions. Under these conditions all three human isolates were reduced in growth over the term of the experiment in the presence of BA and they showed a doubling time (DT of ca. 240 mins). Similarly *Lb. plantarum* strains O5, O13, C9O4 (from table olives) and 21B (from sourdough) all showed reduced DT (270, 240, 240, and 300 mins respectively) in the presence of porcine gallbladder bile (Fig. 1b). These DTs were similar under aerobic conditions (Figure S1). These data suggest that those strains of slower growth may have the ability to metabolize bile acid, a function previously limited to gut associated commensal bacteria. Conversion to more hydrophobic moieties may reduce bacterial growth and this may be an advantage in the GIT. For this reason, the specific ability to metabolize BA was investigated for all of the *Lb. plantarum* strains irrespective of origin.

#### Bacterial alteration of bile acid profile: BSH deconjugation activity

**Figure 2:**
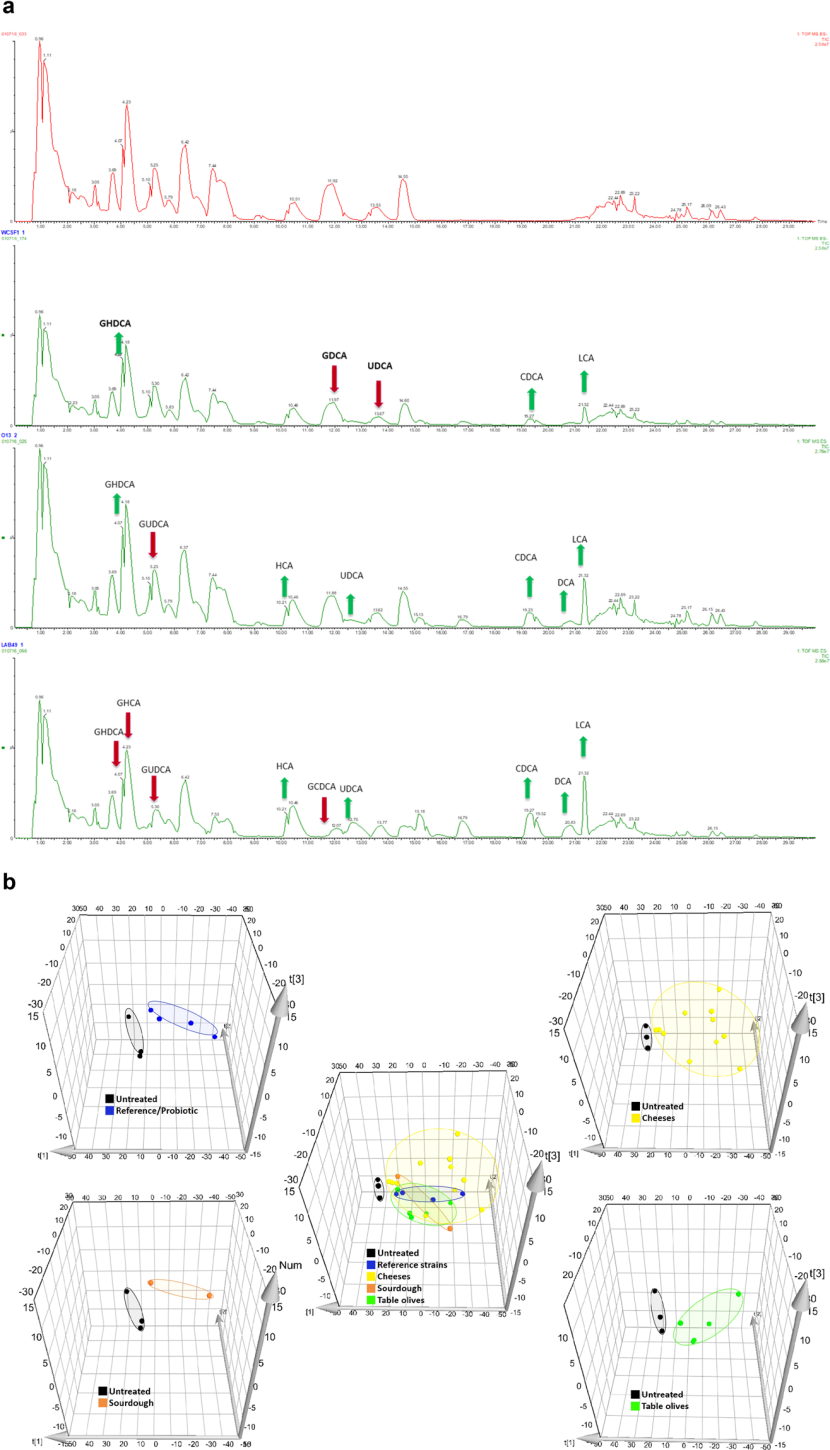
Food associated *Lb. plantarum* strains show differential bile acid metabolism ability. (**a**) UPLC TMS chromatograms showing representative bile acid moiety alterations by food-borne *Lb. plantarum* strains designed by factor of changes (FC). FC was calculated by comparing treated with untreated sample mean values for each bile acid alteration. From the top: Untreated sample, Human *Lb. plantarum* WCFS1 treated sample, food-borne *Lb. plantarum* O13 treated sample; food-borne *Lb. plantarum* LAB49 treated sample. (**b**) Principal Component Analysis (PCA) analysis (generated using MassLynx Software V4.2 SCN943 (WATERS Corporation, USA)) representing untreated and *Lb. plantarum* treated bile acid adjustments as follows: *Lb. plantarum* strains grouped by different origin: reference/probiotics (blue), cheeses (yellow), sourdough (orange), table olives (green).

**Figure 3:**
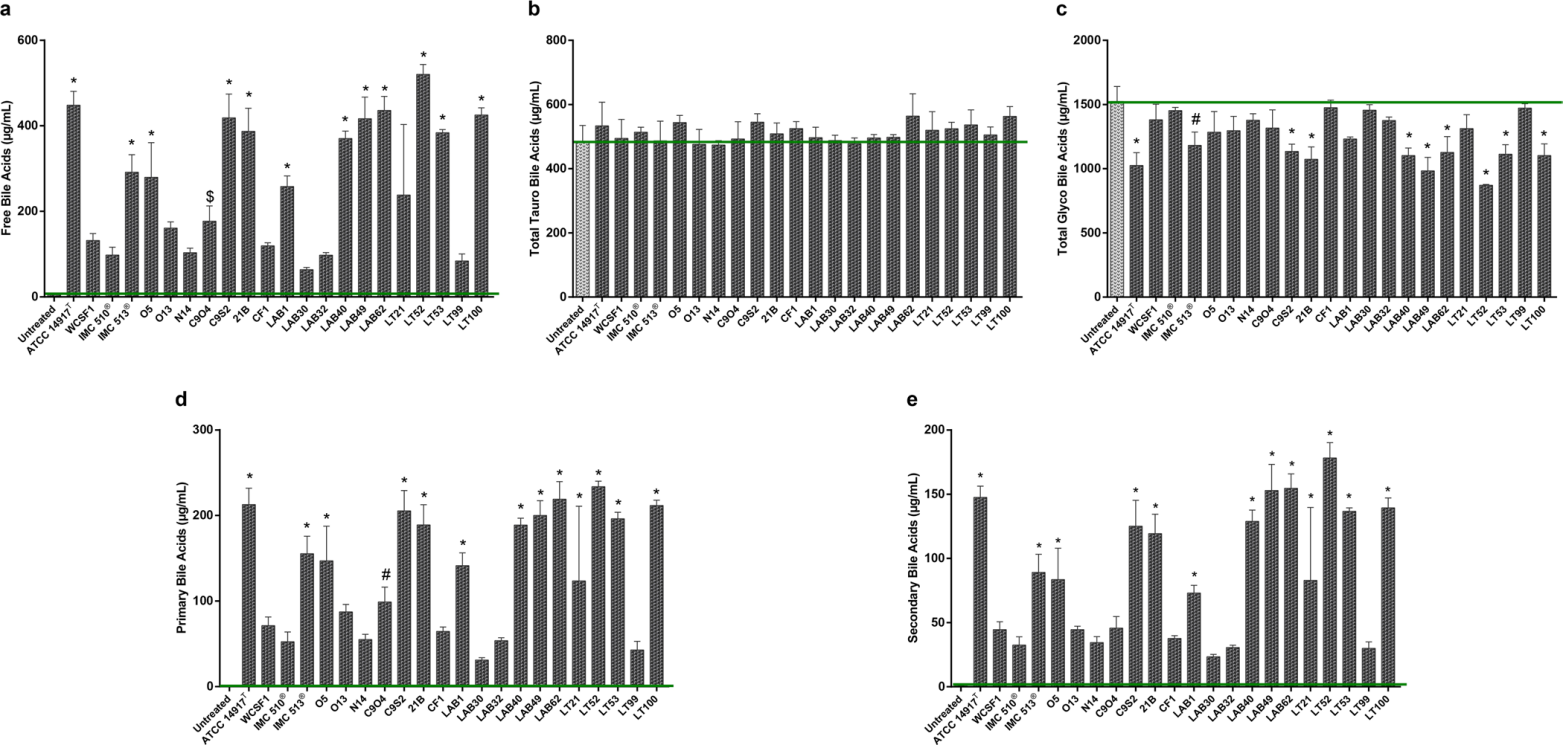
Alteration of bile acid profile by *Lb. plantarum* strains. Assessed by UPLC Q-TOF-MS^E^ in co-incubation supernatants. Green bars show the basal level of bile acid in untreated porcine bile. (**a**) Free bile acids. (**b**) Total Tauro-conjugated bile acids (**c**) Total Glyco-conjugated bile acids. (**d**) Total levels of Primary bile acids (**e**) Total levels of Secondary bile acids. All data are represented as mean ± SD; Statistical analysis were performed by One-Way Anova followed by Bonferroni’s multiple comparisons post hoc test (^$^p < 0.05, ^#^p < 0.01 and *p < 0.001).

**Figure 4:**
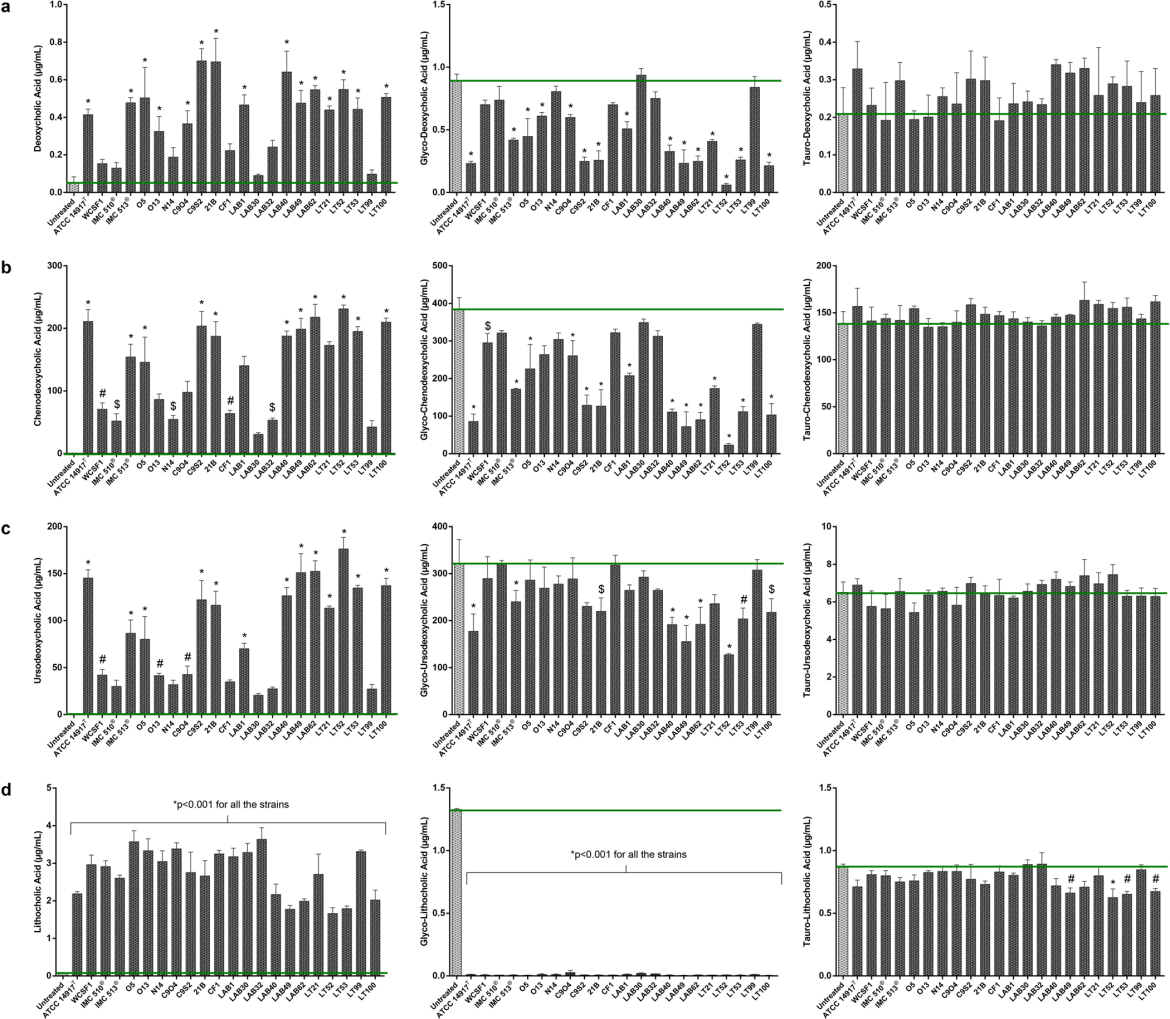
BSH activity of *Lb. plantarum* strains. free, glycoconjugated and tauroconjugated forms of (**a**) Deoxycholic acid, (**b**) Chenodeoxycholic acid, (**c**) Ursodeoxycholic acid and (**d**) Lithocholic acid as representative bile acids moieties showing the high glyco-specific production of unconjugated bile acids compared to the basal level in untreated porcine bile (green bars). Data are presented as means ± SD. Statistical analysis were performed by One-Way Anova followed by Bonferroni’s multiple comparisons post hoc test (^$^p < 0.05, ^#^p < 0.01 and *p < 0.001).

Targeted metabolomics was applied to all 22 strains in order to determine their BA deconjugation ability or BSH activity in which, more than thirty bile acid moieties were assessed (Table S1). BAs derived from the *in vitro* co-incubation assay were extracted through a liquid-liquid extraction procedure (for details see Supplementary Information). Following the method developed by Joyce and coworkers^21^, the efficiency of BAs extraction was assessed by using 2 deuterated internal standards that ensure the correct BA identification and the high linearity of the calibration curves for each moiety allowed facilitated reliable quantification to ng level (Figure S2). UPLC chromatograms showed a clear ability of food-borne *Lb. plantarum* strains to alter bile acid profiles as shown in Fig. 2a, in which it is clear that some unconjugated bile acids are generated (green arrows). Principal Component Analysis (PCA) for all BAs (Fig. 2b) confirm that microbe-bile co-incubations showed altered BA moieties since these groups separated, and were distinct, from control reactions indicating that all of the strains have the ability to deconjugate bile salts, even though they are not directly isolated from the gut environment (Fig. 3). Relative to human gut and saliva associated strains some of the food isolated *Lb. plantarum* strains were equally efficient in their ability to generate free bile acids including secondary BAs DCA, LCA and UDCA albeit to different extents. Moreover, all of the strains show a clear glyco-specific deconjugation activity, while the levels of tauroconjugated bile acid is not significantly altered (Fig. 4). Interestingly when strains were examined for the presence of BSH alleles, the number of BSHs carried by these strains varied in number (from 1-4 BSHs) and in length (data not shown). Their level of activity does not appear dependent on the presence of a specific BSH allele.

**Figure 5:**
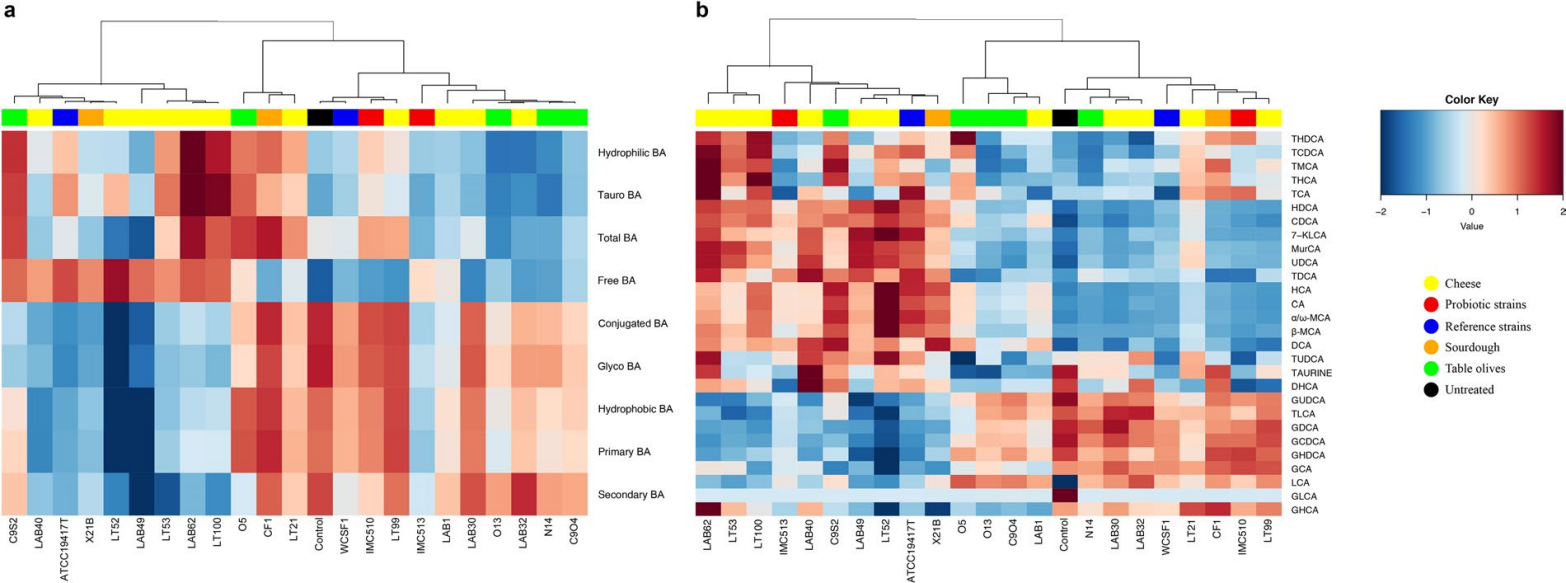
Bile salt hydrolase activity of bacteria strains from different origins. Heat plot indicates alterations to bile acid classes (**a**) and to individual BAs (**b**). Heatmaps were generated using RStudio software, version 1.2.1335 (http://www.rstudio.com).

The deconjugation strength of isolates to generate free BA is as follows: ATCC14917 ≥ C9S2 ≥ LT52 ≥ LT100 ≥ LAB62 ≥ LAB49 ≥ LAB40 ≥ C9O4 > IMC513 > O5 > LAB1 > LT21 > WCFS1 ≥ N14 ≥ CF1 ≥ LAB32 > Lab30. These data indicate that slower growth may not be due to the degree of deconjugation but rather to the levels of specific BAs generated. In this regard, the UPLC Q-TOF-MS^E^ method detected a high production of some free bile acids, specifically DCA, CDCA, UDCA, and LCA, all of them generated from glyco-deconjugation activity (Fig. 5). Variation in bile acid deconjugation profiles indicate selective modifications in a strain-dependent manner. In particular, all strains clearly showed different abilities to generate levels of DCA, CDCA, UDCA, with IMC513 representing the most efficient strain among human associated strains (Fig. 4). Similar differences in BSH activity are reported also for food-associated strains of the same origin. Indeed, the two sourdough associated strains, 21B and CF1 showed distinct activity to generate different levels of DCA, CDCA and UDCA (Fig. 4). A similar effect was evident for raw-milk cheeses strains, where LAB30, LAB32 and LT99 displayed lower activity compared to all of the other strains with same origin, confirming no relationship between type of food and specific BSH activity. Nevertheless, it is important to underline the significant BSH activity and resultant liberation of free bile acids such as DCA, CDCA, UCDA and LCA, as a widespread property among all food-associated isolates in this study.

## Discussion

In order to survive in the human GI tract microorganisms must tolerate and survive numerous environmental insults including variations in pH, low oxygen levels, nutrient limitation and high osmolarity. In terms of bacterial survival in the gut environment, it is necessary to evaluate the ability of potential probiotic strains to endure bile acid-related stress^49,50^. Exposure to bile represents a serious challenge in the gut. Bile is a digestive fluid that emulsifies lipids but when highly hydrophobic and in large quantities it can affect also alter cell membranes, therefore bile tolerance is an important property for both microbial survival and GIT colonization^4^. Bile plays a fundamental role in host specific^51^ and non-specific^52^ defense mechanisms and the magnitude of this inhibitory effect is strongly influenced by BA concentration^53^. In the human GI tract, the mean bile concentration is believed to be 0.3% w/v, a concentration considered as critical and sufficient to screen for bile tolerance and resistance^54^. All of the food associated *Lb. plantarum* strains remained viable in the presence of 1.8% bile and only three strains (*Lb. plantarum* ATCC14917, CF1 and LT99) showed any inhibition of growth when exposed to 3.6% bile. The BA concentration in the gut is not static, but changes over time as well as regionally in the intestine. This is particularly evident after a meal, where bile salt concentrations can increase sharply up to *ca*. 15 mmol/L in the duodenum, in the ileum, the concentration falls below 4 mmol/L due to active ileal BA re-absorption^51^. Considering that BAs form micelles with phospholipids, cholesterol and fatty acids (whole bile) they have lower antibacterial activity than artificial solutions of pure BAs^48^ we also investigated bile tolerance by incubating all the strains with 0.5% w/v porcine bile (of gall-bladder origin). All of the strains were able to tolerate porcine bile, under both anaerobic and aerobic conditions and they showed similar growth in either presence or absence of bile (Figs. 1b and SI, Figure S1b).

These data support the adaptation ability of *Lb. plantarum*, a versatile lactic acid bacterium that occupies a range of environmental niches including dairy, meat and many vegetable fermentations as well as the human GI tract^55^. *Lb. plantarum* has one of the largest genomes (3.3 Mb) characterized among *Lactobacilli*, this may reflect its ecological flexibility for occupancy of several environmental niches^56^. The *Lb. plantarum* WCFS1 genome^57^ is predicted to contain four BSH related genes, annotated as *bsh*1 to *bsh*4, they are spread throughout the genome, and *bsh*1 appears solely responsible for the BA metabolism ability of *Lb. plantarum* strains.

Our data points towards the ability of all of the strains applied to this study, both food-borne and human isolates to survive one of the main gastro-intestinal stresses, and combined with our previous studies in an *in vitro* GI model^46,47^ we suggest that all of the strains should be viable on reaching the duodenum and in GI transit. Microbial bile tolerance is a recognized criteria for probiotic strain selection, and BSH activity is a desirable property when selecting a strain for use as a dietary adjunct^10,13,58^. In this regard, microbial BSH activity is the first step in bile salt biotransformation in the gut. BSH activity is considered a common feature of gut microorganisms that is distributed across the major phyla of bacteria in the gut as well as the gut Archea^8^. Moreover, this activity has been recognised as important in mediating a microbe-host dialogue that functionally regulates host lipid metabolism and plays a profound role in cholesterol metabolism^16,59–61^. Microbial BA modulation and metabolism has been shown to significantly impact on host physiology^13^, and they have been linked to alterations in blood cholesterol levels^19,36,62,63^ with the potential to regulate body weight and lipid metabolism in mice. This highlights microbial BA alterations as a novel strategy to influence weight gain in the host^21,61^.

To date, there is a scarcity in publications where BA metabolism by bacteria isolated from fermented foods has been examined. In the present study, we determine that food-borne *Lb. plantarum* strains can metabolize BAs, albeit to different extents and at levels that are similar to those reported from human isolates. Thus, the idea that BSH activity is strictly limited to intestinal resident or to tourist pathogenic strains should be reconsidered. This work highlights strain-specific variation in BAs deconjugation ability among the food isolates (Fig. 5) there is no strict correlation between food origin and BSH activity. Moreover, we demonstrate that the food associated isolates, from this study, are adapted for glyco-specific deconjugation activity, while the ability to act on tauro-conjugated bile salts is not significant (Fig. 3). BSH positive bacteria have different affinities and relative activity against conjugated substrates^4^. The preference to metabolize glycine- over taurine-conjugated BAs is also a feature of *Lb. plantarum* WCFS1. This specificity could represent an evolutionary preference for glycine-conjugated BAs, given their increased prevalence in the human GIT and their proposed higher toxicity in comparison to taurine-conjugated BA^64^. Although the precise mechanism by which BA amidates are selected by BSHs is uncharacterized, the differential affinity for a glyco- or tauro-conjugated BA specific substrates, suggests that BSH hydrolysis could be of importance for bacterial survival *in vivo*.

The *Lb. plantarum* food isolates, from this study, support accumulation of free bile acids DCA, CDCA, UCDA and LCA while maintaining tauro-conjugated BA as relatively untouched (Figs. 4 and 5). These free BAs are ligands to influence the activity of certain host receptors, expressed in many organs and tissues (liver, gallbladder and intestine) including the nuclear bile acid receptor farnesoid X receptor (FXR) and Vitamin D receptor (VDR) indicating the dual digestive and signaling role for BAs in the host^16,59,60,65^. These interactions with can influence energy metabolism, liver function and intestinal health, they can also improve insulin sensitivity and weight loss^16,21,66^. The FXR is preferentially stimulated by the specific free and secondary BAs that our strains facilitate to accumulate. FXR activity is considered protective against colorectal cancer suggesting that BA deconjugation may enhance the anti-cancer effects of FXR^67^. Activation of TGR5, by taurine conjugated secondary BAs: TDCA and TLCA, conjugates that remain intact in the presence of the *Lb. plantarum* isolates reported here, BA activated TGR5 can increase browning of white adipose tissue and induce thyroxine production to increase energy expenditure, thereby influencing diet-induced obesity^68^. BAs are also proposed to contribute to regulation of whole-body glucose and lipid metabolism and therefore body weight^61^. It has been universally recognized that bacterial modulation of the BA pool, especially by the modulation of FXR and TGR5 signaling, plays a fundamental role in regulating host homeostasis as well as lipid and cholesterol host metabolism^9,61^. In addition, it has been found that modulation of BA profiles is also related to the peripheral circadian clock, an interesting aspect that may be correlated to food intake and obesity^69^. Thus, microbial BSH activity could be considered a beneficial property that may find importance for novel preventive and therapeutic strategies for conditions associated with BA dysbiosis including cirrhosis, fatty liver disease, cholestasis, colorectal cancer, certain types of *Helicobacter*-associated cancer as well as obesity and related metabolic diseases.

In conclusion, this is one of the first studies to investigate a collection of *Lb. plantarum* strains, isolated from fermented foods for their ability to metabolize BAs, as an adaptation mechanism to persist in the gut environment. All of the food-borne *Lb. plantarum* strains examined here were shown to have BSH activity to a similar level to human isolates even though they are not gut-associated. Variation in BA deconjugation profiles indicated subtle selective modifications in a strain-dependent manner with a clear and significant ability to utilise glyco-conjugated BAs as specific substrates. BSH activity in all isolates led to the accumulation of free BAs such as DCA, CDCA, UCDA and LCA, all of these are recognized as BAs signalling molecules to contribute to regulation of whole-body glucose and lipid metabolism.

From a host perspective, this microbial activity can potentially lead to alterations in host metabolic processes, by modulating the BA pool and host receptor signalling. In addition, the experimental evidence reported in this study, suggests that we must reconsider the concept that BSH activity is an exclusive feature of gut-associated microbes alone. Overall, this work highlights the beneficial contribution of fermented foods through their microbial components (as *Lb. plantarum* species) to the BA metabolism and their potential to modulate host metabolic processes. Finally, this study highlights that food-borne microbes are equipped, somewhat, for gut survival. Considering that, microbes are commonly introduced with a foodstuff that may facilitate their propagation in the GI tract, our data indicate the importance of future studies to monitor and track food-associated non-pathogenic microorganisms through GI transit, in order to determine transient from stable colonization and interaction with the host as well as monitor their outgrowth and genetic adaptation to both food and the gut environments.

## Methods

### Bacterial strains

A collection of 22 strains of *Lactobacillus plantarum* belonging to our laboratory culture collection at the University of Teramo, were investigated in this study. All the strains were originally isolated from fermented foods as raw-milk cheeses (11 strains), table olives (5 strains) and sourdough (2 strains) with the exception of *Lb. plantarum* WCFS1 and ATCC14917 and two strains with documented probiotic activities, IMC 510, and IMC 513 (kindly provided by SYNBIOTECH, Camerino, Italy), included in the study as reference strains (Table 1). All the strains were previously characterized as *Lb. plantarum* species and tested for several properties including their potential ability to survive in GI transit^46,47^. They were routinely grown under microaerophilic conditions using de Man, Rogosa and Sharp (MRS) medium (Oxoid) at 37°.

**Table 1:** *Lb. plantarum* strains investigated in this study including their food of origin and source.

Strain	Origin	Source
WCFS1	Human saliva	Reference strain, UNITE Collection
ATCC14917	Pickled cabbage	Reference strain, UNITE Collection
IMC510, IMC513	Human gut	Probiotic strain, UNICAM
O5, O13, N14, C9O4, C9S2	Table olives	Laboratory isolates
21B, CF1	Sourdough	Laboratory isolates
LAB1,LAB30,LAB32,LAB40,LAB49,LAB62	Raw-milk cheeses	Laboratory isolates
LT21, LT52, LT53, LT99, LT100	Raw-milk cheeses	Laboratory isolates

### Bile salt resistance

A spectrophotometric assay was carried out in order to investigate microbial bile salts tolerance, by using a modified method described by Gilliland and coworkers^51^. Isolates were grown in MRS broth at 37 °C to obtain overnight cultures. Then 0.5 ml from serial dilutions were added to MRS broth to obtain an initial inoculum concentration of 10^3^ CFU/ml and all strains were co-incubated in MRS broth at 37 °C for 24 hours with increasing concentrations of bile salts (OXOID) (0%, 0.30%, 0.60%, 1.8%, 3.6% w/v). After 24 hours of incubation, microbial growth was assessed turbidimetrically by measuring optical density at 600 nm (Fig. 1a).

### Bile tolerance

Microbial bile tolerance was assessed for each strain by monitoring growth in presence of gallbladder porcine bile. All the strains were incubated in MRS broth with porcine gallbladder bile (0.5% w/v) both in aerobic and anaerobic conditions, and O.D. at 600 nm was taken every hour for 24 hours to follow bacterial growth (Figs. 1b and S1 Figure S1b).

### Bile salt hydrolase deconjugation activity

The bacterial ability to deconjugate bile acids was investigated by performing a coincubation *in vitro* assay followed by Ultra Performance Liquid Chromatography coupled with an electrospray ionization Quadrupole Time-Of-Flight Mass Spectrometry operating in MS^E^ mode (UPLC Q-TOF-MS^E^). Briefly, all *Lb. plantarum* strains were coincubated for 90 minutes in porcine gallbladder bile (0.5% w/v) and then a liquid-liquid extraction was carried out to recover all bile acids (Table S1). UPLC Q-TOF-MS^E^ was performed to analyse the BAs profile according to Joyce and coworkers^21^. *Lb. casei* NCDO161 was used as a *bsh* negative control in the study (Figure S3). For experimentally details see SI Materials and Methods^70,71^.

### Statistical analysis

Data from three biological replicates, reported as mean values with SD, were statistically analysed by one-way analysis of variance (ANOVA) and Student t test. Differences between strain means were tested for significance by Bonferroni’s multiple comparison test using PRISM 7.0 (GRAPHPAD Software Inc., La Jolla, CA). Principal Component Analysis of microbial alteration of BAs profile were performed by using MassLynx Software V4.2 SCN943 (WATERS Corporation, USA).

## Supplementary information

Supplementary data.
