# Supplementary material for: Beneficial bile acid metabolism from Lactobacillus plantarum of food origin
Source: Sci Rep. 2020 Jan 24;10:1165. doi: 10.1038/s41598-020-58069-5 (PMC6981223; doi:10.1038/s41598-020-58069-5)
Supplement: Supplementary file 1 — Supplementary data. [file 41598_2020_58069_MOESM1_ESM.pdf]

Beneficial bile acid metabolism from *Lactobacillus plantarum* of food origin

Roberta Prete [1,2], Sarah Louise Long[2,3], Alvaro Lopez Gallardo[2], Cormac G. Gahan[2,3,4], A. Corsetti[1] and Susan A. Joyce [2,5*]

**corresponding author*
s.joyce@ucc.ie

[1] University of Teramo, Faculty of Bioscience and Technology for Food, Agriculture and Environment, 64100, Via Balzarini 1, Teramo, Italy

[2] APC Microbiome Ireland, University College Cork, Cork, Ireland

[3] School of Microbiology, University College Cork, Cork, Ireland

[4] School of Pharmacy, University College Cork, Cork, Ireland

[5] School of Biochemistry and Cell Biology, University College Cork, Cork, Ireland.

Supplementary Information

SI Materials and Methods

Chemicals and Reagents.

Standard conjugated bile acids and bile acids were purchased from Sigma Aldrich or from Steraloids, they are listed in Table S1. Deuterated cholic acid (D -2452) and deuterated chenodeoxycholic acid (D -2772) were purchased from CDN Isotopes Inc. HPLC-grade methanol, acetonitrile, water, ammonium acetate, ammonium formate, ammonium hydroxide, formic acid, and acetic acid and water were obtained from Fisher Scientific. Standards were constituted as 1 mg/mL. Stock solutions of individual bile acids were prepared in water: MeOH (1:1) and combined to a final volume of 1.0 mL concentration of 40 µg/mL for each. Subsequent dilutions were made as required.

Bile Acid Extractions.

Bile acids were extracted from bacteria-bile coincubation supernatants added to 50% ice-cold methanol. The extract was mixed and then was centrifuged at 16000 × g for 10 min at 4 °C. The supernatant was retained and further extracted by the addition of acetonitrile with 5% formic acid. The resultant supernatant was dried under vacuum and reconstituted in water: MeOH (1:1) prior to to LC-MS analysis. The extracted bile acids were resuspended in 150 ml of ice cold 50% MeOH.

UPLC Q-TOF-MS^E.

UPLC Q-TOF-MS^E was performed using the method developed by Joyce *et al*., [21]. Briefly, 5 µl of extracted bile acids were injected onto Acquity UPLC BEH C18 analytical column T3 Acquity column (Waters Corp.) and eluted using a 25-min methanol-acetonitrile gradient at a flow rate of 400 µL/min and column temperature of 45 °C.

Samples were analyzed using an Acquity UPLC system (Waters Ltd.) coupled online to an Xevo G2 QTof LCT Premier mass spectrometer (Waters MS Technologies, Ltd.) in negative electrospray mode with a scan range of 50 - 1,000 m/z. Bile acids ionize strongly in negative mode, producing a prominent [M-H] negative ion. Capillary voltage was 2.4 Kv, sample cone was 35 V, desolvation temperature was 350°C, source temperature was 120 °C, and desolvation gas flow was 900 L/h.

Principal components analysis (PCA) was performed in Targetlynx (Waters). Furthermore a template of defined known masses was applied to allow the detection of bile acid only. The groups (types) are shown in different colours, and the separation of the groups is easily visible (Figure 2B). Each analyte was identified according to its mass and retention time. Standard curves were performed using known bile acids, and each analyte was quantified according to the standard curve and normalized according to the deuterated internal standards (Figure S2).

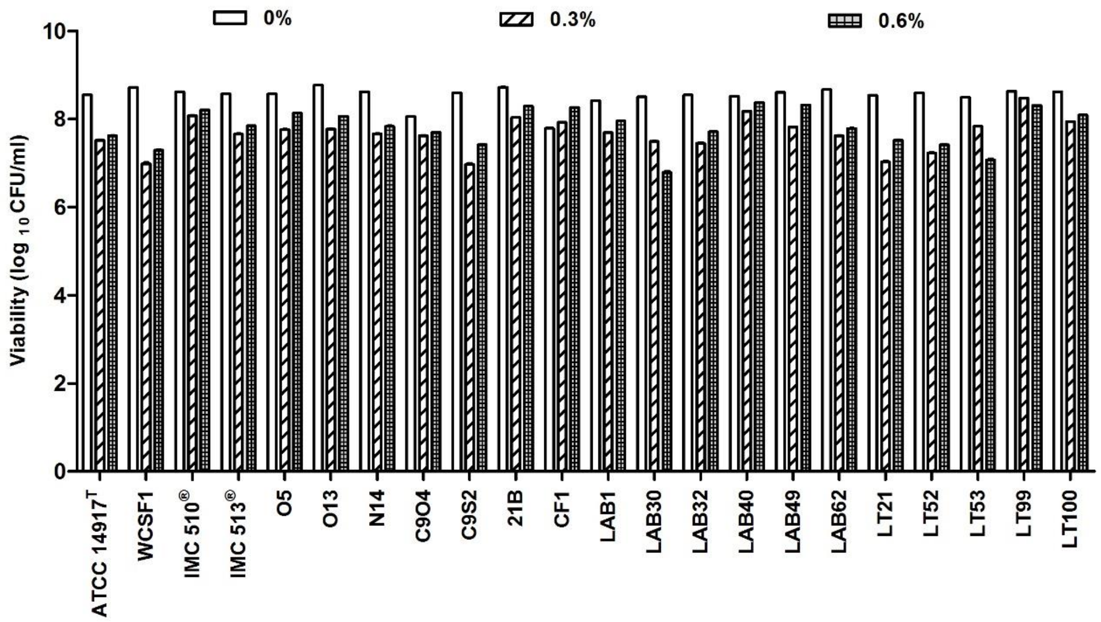

Figure S1a. Cell viability of *Lb. plantarum* strains to bile salts exposure. Data are shown as mean values and bars indicate the standard deviation (SD) obtained from three replicates. One-Way Anova followed by Bonferroni's multiple comparisons post hoc test showed no significant differences.

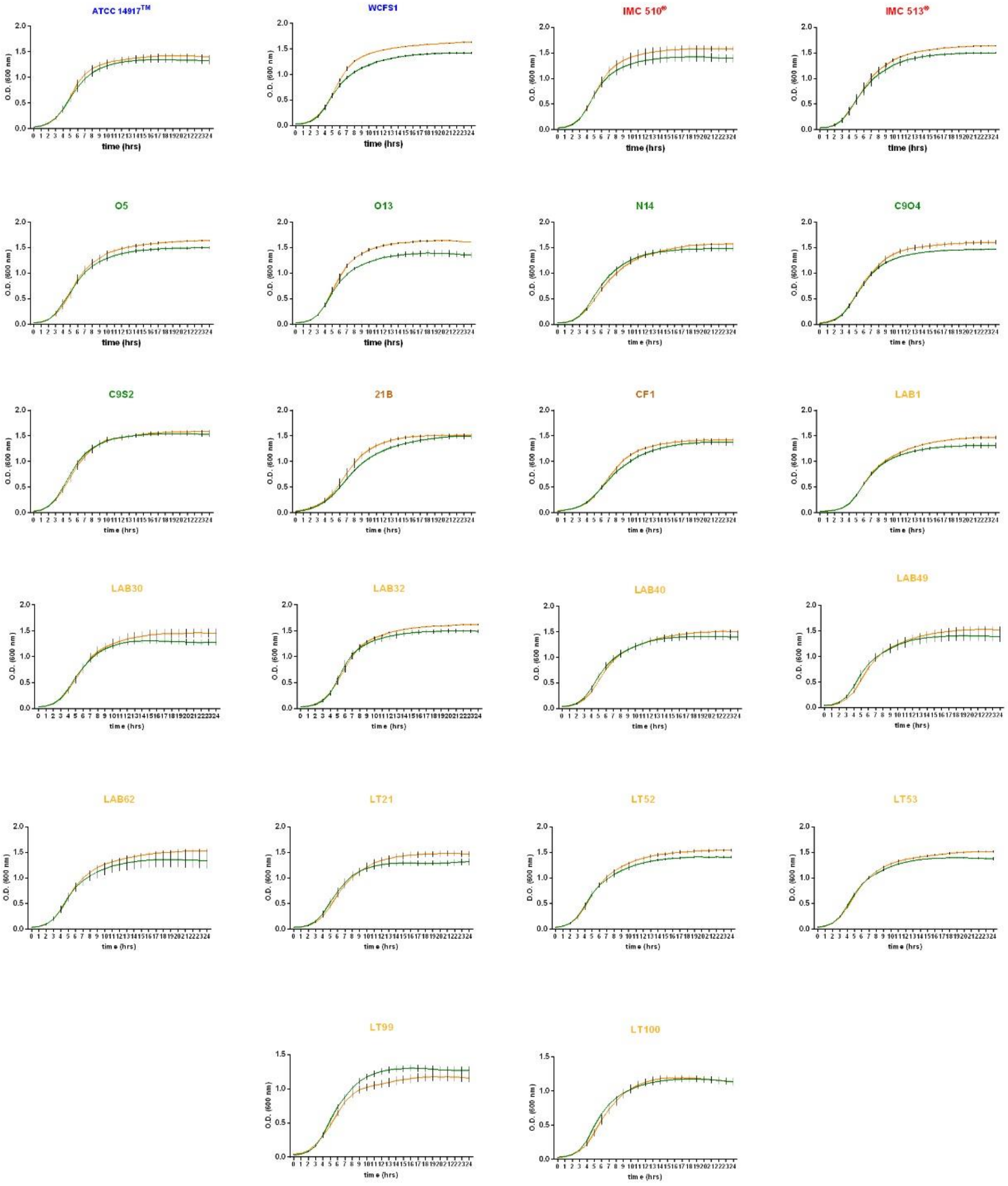

Figure S1b. Aerobic bile tolerance growth curves (orange line means MRS broth, green lines means MRS broth with porcine bile (0.5% w/v). Data are reported as means with SD from three biological replicates.

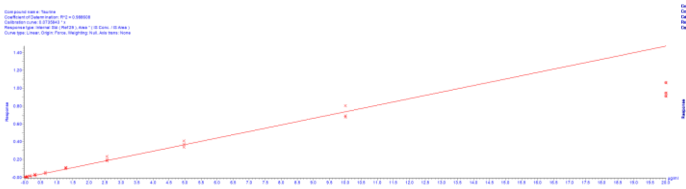

Taurine

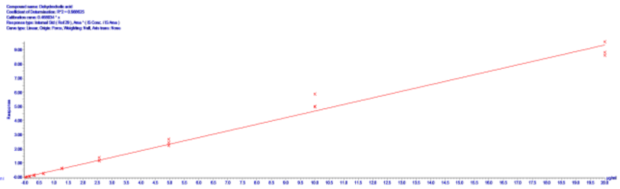

Dehydrocholic acid

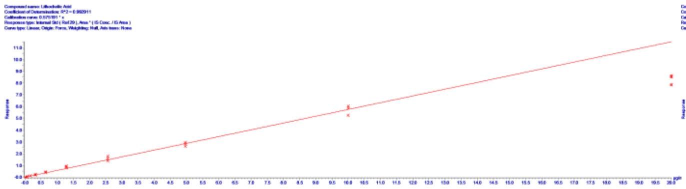

Lithocholic acid

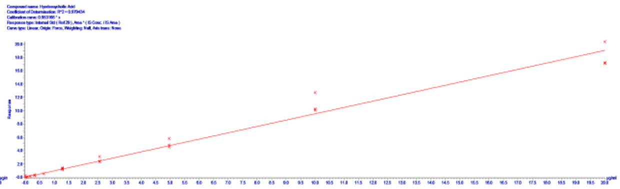

Hyodeoxycholic acid

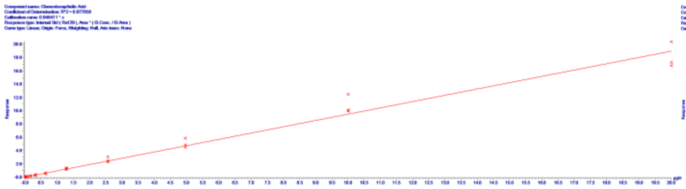

Chenodeoxycholic acid

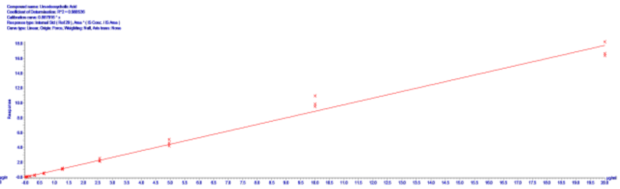

Ursodeoxycholic acid

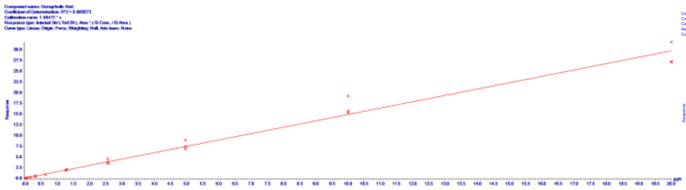

Deoxycholic acid

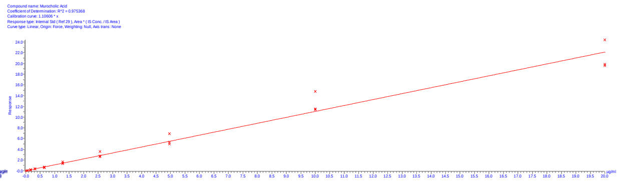

Murocholic acid

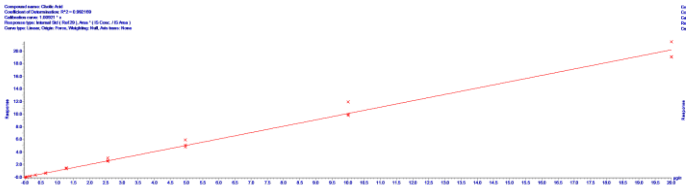

Cholic acid

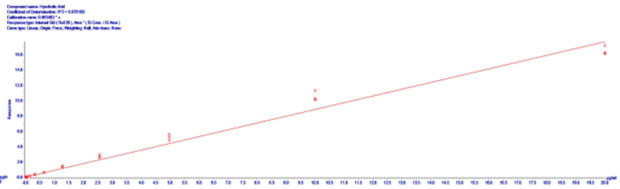

Hyocholic acid

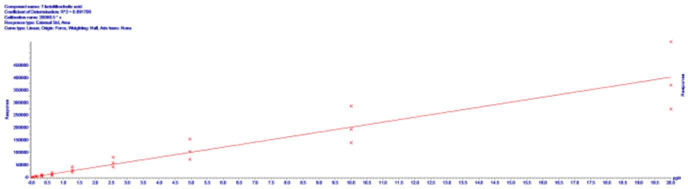

7-ketolithocholic acid

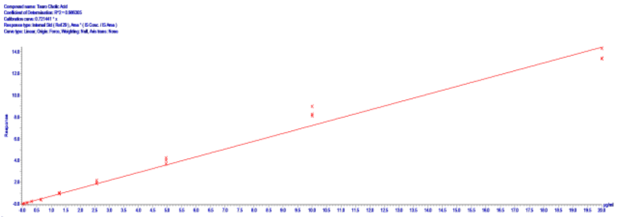

Tauro Cholic acid

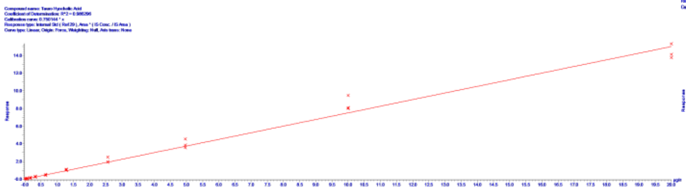

Tauro Hyocholic acid

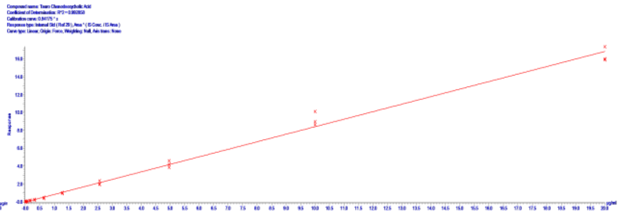

Tauro Chenodeoxycholic acid

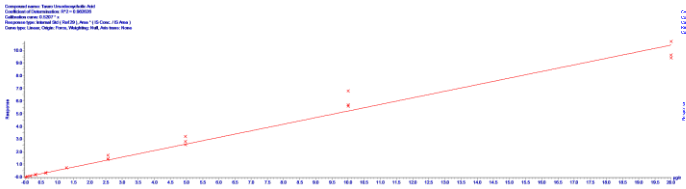

Tauro Ursodeoxycholic acid

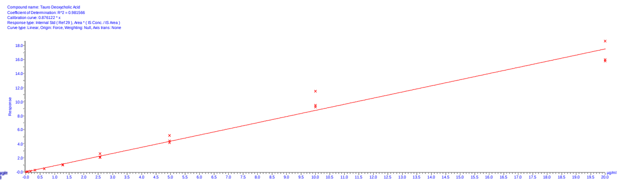

Tauro Deoxycholic acid

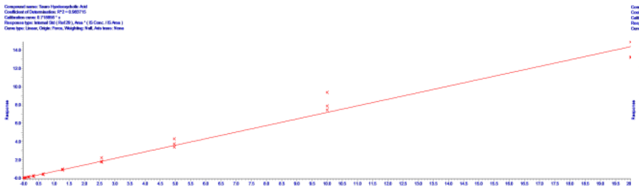

Tauro Hyodeoxycholic acid

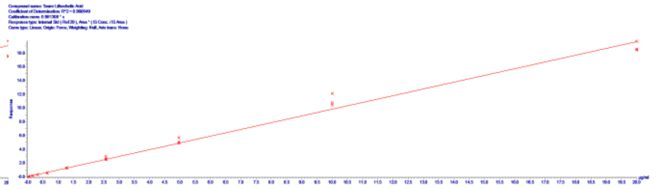

Tauro Lithocholic acid

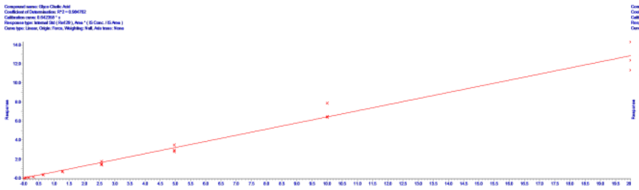

Glyco Cholic acid

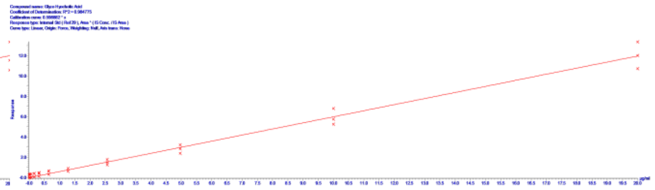

Glyco Hyocholic acid

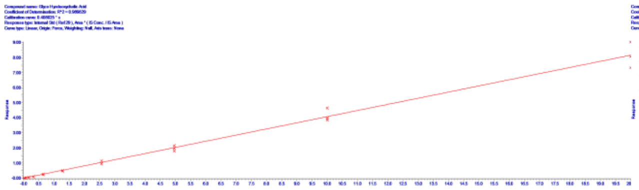

Glyco Hyodeoxycholic acid

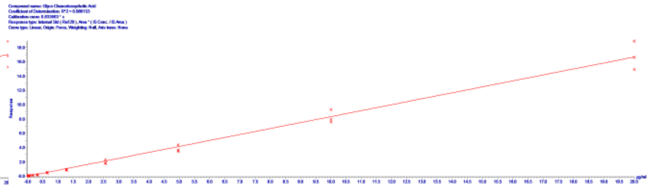

Glyco Chenodeoxycholic acid

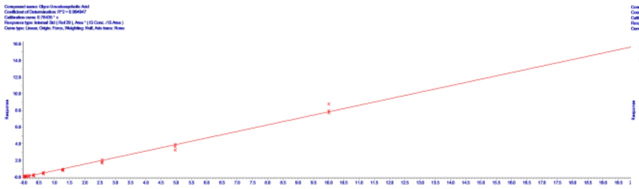

Glyco Ursodeoxycholic acid

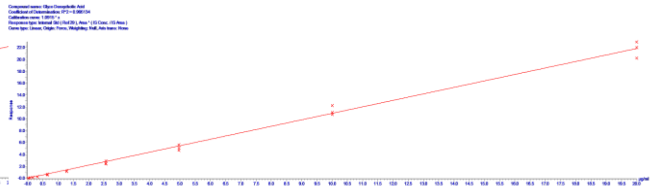

Glyco Deoxycholic acid

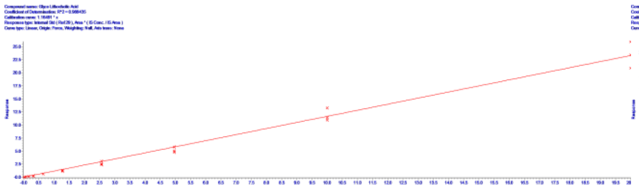

Glyco Lithocholic acid

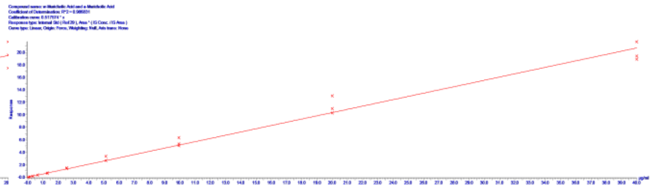

ω-Muricholic acid and α-Muricholic acid

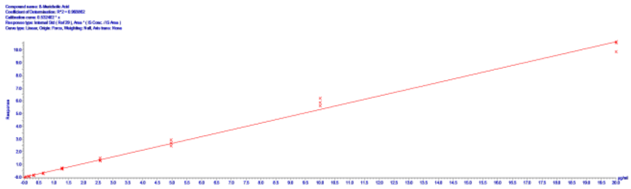

Tauro β-Muricholic acid

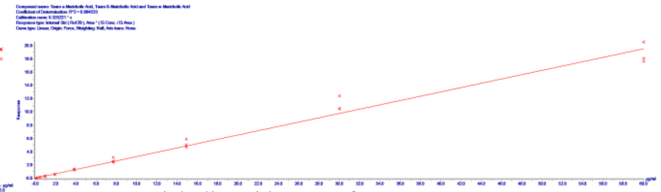

Tauro α-Muricholic acid, Tauro β-Muricholic acid and Tauro ω-Muricholic acid

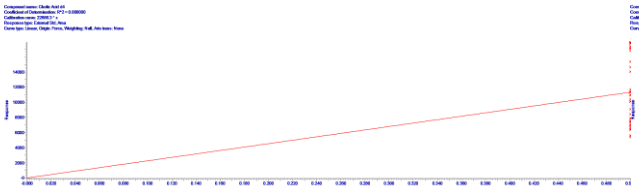

Cholic acid d4 (*internal standard*)

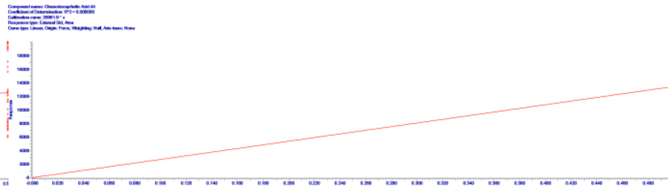

Chenodeoxycholic acid d4 (*internal standard*)

Figure S2. Standard curves used to quantify each bile acid moiety alterations.Standards dilutions were utilized to create standard curves for each analyte examined (n=3). Internal deuterated standards facilitated assessment of extraction efficiency and normalization between samples.

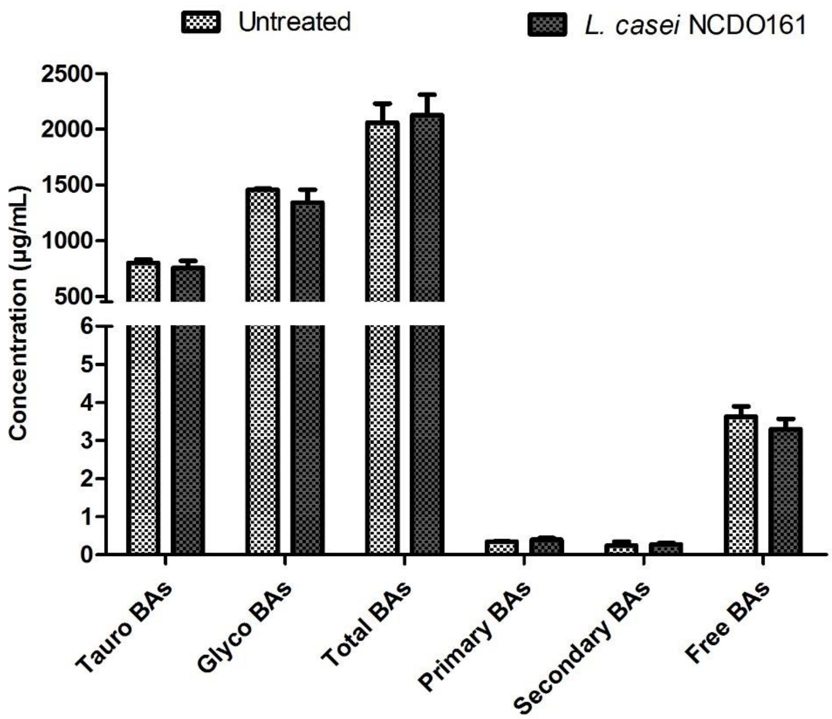

Figure S3. *Lactobacillus casei* NCDO161 alteration of bile acids (BAs) was examined a as a *bsh* negative control for the study. Bile acid signatures were assessed by UPLC Q-TOF-MS^E and data are reported as mean values with standard deviation (SD). Statistical comparisons were performed using both Student t test and Two-way ANOVA with Bonferroni's post hoc test did not reveal any significant differences when compaisons were made with the untreated sample (p = 0.9758 and p = 0.5650).

Table S1. Bile acid moieties analyzed by UPLC Q-TOF-MS^E in this study together with their neutral formula, mass, standard curve R^2 value and range detected.

Analyte	$[MW]^{-H}$	Formula	R^2 value	Range (µg/ml)
Taurine	124.0068	$C_2H_7NO_3S$	0.99	0.0064-20.00
Cholic Acid	407.2797	$C_{24}H_{40}O_5$	0.99	0.0064-20.00
Chenodeoxycholic Acid	391.2848	$C_{24}H_{40}O_4$	0.98	0.0064-20.00
Lithocholic Acid	375.2899	$C_{24}H_{40}O_3$	0.99	0.0064-10.00
Deoxycholic Acid	391.2848	$C_{24}H_{40}O_4$	0.98	0.0064-20.00
Dehydrocholic Acid	401.2328	$C_{24}H_{34}O_5$	0.99	0.0064-20.00
Ursodeoxycholic aAid	391.2848	$C_{24}H_{40}O_4$	0.99	0.0064-20.00
Hyocholic Acid	407.2797	$C_{24}H_{40}O_5$	0.98	0.0064-20.00
Hyodeoxycholic Acid	391.2848	$C_{24}H_{40}O_4$	0.98	0.0064-20.00
7-Ketolithocholic Acid	389.2692	$C_{24}H_{38}O_4$	0.89	0.0064-20.00
Murocholic Acid	391.2848	$C_{24}H_{40}O_4$	0.98	0.0064-20.00
Taurocholic Acid	514.2838	$C_{26}H_{45}NO_7S$	0.99	0.0064-20.00
Taurochenodeoxycholic Acid	498.2889	$C_{26}H_{45}NO_6S$	0.99	0.0064-20.00
Taurolithocholic Acid	482.2940	$C_{26}H_{45}NO_5S$	0.99	0.0064-20.00
Taurodeoxycholic Acid	498.2889	$C_{26}H_{45}NO_6S$	0.98	0.0064-20.00
Taurohyocholic Acid	514.2838	$C_{26}H_{45}NO_7S$	0.99	0.0064-20.00
Tauroursodeoxycholic Acid	498.2889	$C_{26}H_{45}NO_6S$	0.98	0.0064-20.00
Taurohyodeoxycholic Acid	498.2889	$C_{26}H_{45}NO_6S$	0.98	0.0064-20.00
Glycocholic Acid	464.3012	$C_{26}H_{43}NO_6$	0.98	0.0064-20.00
Glycochenodeoxycholic Acid	448.3063	$C_{26}H_{43}NO_5$	0.99	0.0064-20.00
Glycolithocholic Acid	432.3114	$C_{26}H_{43}NO_4$	0.99	0.0064-20.00
Glycodeoxycholic Acid	448.3063	$C_{26}H_{43}NO_5$	1.00	0.0064-20.00
Glycohyocholic Acid	464.3012	$C_{26}H_{43}NO_6$	0.98	0.0064-20.00
Glycoursodeoxycholic Acid	448.3063	$C_{26}H_{43}NO_5$	0.99	0.0064-20.00
Glycohyodeoxycholic Acid	448.3063	$C_{26}H_{43}NO_5$	0.99	0.0064-20.00
α-Muricholic Acid	407.2797	$C_{24}H_{40}O_5$	0.99	0.0064-20.00
β-Muricholic Acid	407.2797	$C_{24}H_{40}O_5$	0.99	0.0064-20.00
ω-Muricholic Acid	407.2797	$C_{24}H_{40}O_5$	0.99	0.0064-20.00
Tauro α-Muricholic Acid	514.2838	$C_{26}H_{45}NO_7S$	0.98	0.0064-20.00
Tauro β-Muricholic Acid	514.2838	$C_{26}H_{45}NO_7S$	0.98	0.0064-20.00
Tauro ω-Muricholic Acid	514.2838	$C_{26}H_{45}NO_7S$	0.98	0.0064-20.00
Cholic Acid d4	411.3049	$C_{24}H_{36}D_4O_5$	NA	0.0064-20.00
Chenodeoxycholic Acid d4	395.3099	$C_{24}H_{36}D_4O_4$	NA	0.0064-20.00
